# Contrasting tree growth and soil organic carbon across stand densities in a *Picea crassifolia* plantation: associations with soil nitrogen

**DOI:** 10.3389/fpls.2026.1926241

**Published:** 2026-09-15

**Authors:** Xingpeng Zhao, Xin Jia, Hang Xiang, Dong Lv, Hu Zhao, Erwen Xu, Rong Zhou, Ming Zhao, Mengde Liu, Zhiwen Gao

**Affiliations:** 1 Academy of Water Resources Conservation Forest of Qilian Mountains, Zhangye, Gansu, China; 2 College of Grassland Science, Gansu Agricultural University, Lanzhou, China; 3 Western Carbon Sink Trading Asset Management (Gansu) Co., Ltd., Zhangye, Gansu, China; 4 Climate Bridge (Shanghai) Environmental Technology Co., Ltd., Shanghai, China

**Keywords:** nitrogen-driven, *Picea crassifolia*, plantation, soil organic carbon, stand density

## Abstract

**Introduction:**

Stand density can influence both tree growth and belowground carbon storage in plantations, but density conditions favorable for these two functions may not necessarily coincide.

**Methods:**

We investigated a *Picea crassifolia* plantation in the Qilian Mountains under five stand-density conditions with nominal initial densities of approximately 4,444, 2,500, 2,000, 3,333, and 2,667 stems ha⁻¹. Each treatment comprised three spatially independent replicate stands, with three 50 × 50 m quadrats established within each stand, resulting in nine quadrat-level observations per treatment. Tree growth, leaf and root C, N, and P concentrations, soil physical properties, and soil organic carbon (SOC), total nitrogen (TN), and total phosphorus (TP) contents and densities were evaluated across the 0–80 cm soil profile. Correlation analysis, redundancy analysis (RDA), and exploratory structural equation modeling (SEM) were further used to examine statistical associations among the measured variables.

**Results:**

Tree height, ground diameter, and crown development were greater under the nominal 2,000 and 2,667 stems ha⁻¹ treatments, whereas these conditions did not consistently correspond to greater soil C and nutrient storage. In contrast, the nominal 3,333 and 2,500 stems ha⁻¹ treatments generally showed higher SOC, TN, and TP densities in several soil layers. RDA indicated that TP contributed most strongly to the multivariate differentiation among the measured traits, whereas exploratory SEM identified TN as the strongest statistical correlate of SOC among the measured soil variables.

**Discussion:**

These findings demonstrate contrasting density-associated patterns between aboveground tree growth and belowground carbon and nutrient storage. Under the conditions examined here, density management should therefore consider the balance between tree growth and soil carbon and nutrient functions rather than assume a single universally optimal stand density.

## 1 Introduction

Forest soils represent a major component of terrestrial carbon storage and play an important role in regulating the long-term carbon balance of forest ecosystems. Enhancing soil organic carbon (SOC) stocks is therefore increasingly recognized as an important component of climate-change mitigation and sustainable forest management (Pan et al., 2011; Bossio et al., 2020). Planted forests contribute substantially to terrestrial carbon sequestration, but their belowground carbon storage depends not only on tree growth but also on soil water availability, nutrient cycling, organic matter inputs, and decomposition processes (Hong et al., 2023) Recent large-scale assessments have further demonstrated the growing contribution of planted forests to regional carbon storage. For example, the carbon stock of planted forests in China increased markedly between 1990 and 2020 as a result of both forest-area expansion and stand development (Cheng et al., 2024). These findings highlight that increasing planted-forest area alone is insufficient to explain future carbon-sink capacity and that the structure and management of existing plantations also require greater attention. Identifying manageable stand characteristics associated with greater soil carbon storage is therefore particularly important for plantations in environmentally sensitive and water-limited regions.

Among silvicultural variables, stand density is particularly relevant because it is directly manageable and can simultaneously alter competition for light, water, and nutrients, thereby affecting canopy structure, soil moisture, litter inputs, root distribution, nutrient availability, and tree growth (Dang et al., 2018; Sterck et al., 2021). Stand density also determines how limited resources are partitioned among individual trees and the stand as a whole, meaning that density conditions maximizing individual growth may differ from those favoring stand-level carbon storage. Recent work in water-limited *Larix principis-rupprechtii* plantations has demonstrated that the balance between individual-tree growth and stand carbon storage varies with both stand density and moisture conditions (Wu et al., 2026b). More broadly, silvicultural studies have shown that management for carbon storage can involve trade-offs with other structural objectives rather than producing a single universally optimal stand condition (Pretzsch and Hilmers, 2025). Relationships between stand density and soil carbon are likewise inconsistent among forest ecosystems. Higher stand densities have been associated with greater soil carbon storage in some plantations (Na et al., 2021), whereas relatively low-density conditions have been associated with greater carbon and nitrogen storage in some *Picea crassifolia* forests (Feng et al., 2023). Similar density-dependent differences in soil C, N, and P have also been reported for other plantation species (Sun et al., 2018; Cheng et al., 2023). Moreover, recent density-gradient research in Principis-rupprechtii L. plantations indicates that stand-density effects on labile carbon pools and soil respiration can vary among stand-age classes, further illustrating the context dependence of belowground carbon responses (Liu et al., 2025). These contrasting findings suggest that no universally optimal density can be assumed and that density conditions associated with greater tree growth may not necessarily coincide with those associated with greater belowground carbon storage.

Stand density may be associated with SOC through several interacting pathways. Density-related changes in canopy interception and evapotranspiration can modify soil water availability, which is closely linked to nutrient mobility, plant nutrient uptake, root activity, and microbial decomposition (Cui et al., 2020; Zhai et al., 2024). Root turnover and rhizosphere processes may further influence belowground carbon inputs and SOC stabilization (Jia et al., 2022), while soil nutrient availability, particularly nitrogen (N), is closely coupled with plant carbon allocation and soil carbon retention (Hong et al., 2023). Stand-density effects may therefore involve both carbon inputs and carbon losses: changes in root activity and litter supply can alter substrate inputs, whereas changes in soil temperature, moisture, microbial biomass, extracellular enzyme activity, and respiration can simultaneously modify carbon turnover. Recent plantation studies support this view, showing density-associated variation in labile SOC fractions, microbial biomass carbon, extracellular enzyme activities, and soil respiration, with soil moisture among the important covarying factors (Liu et al., 2025). Consequently, SOC responses to stand density cannot be inferred solely from aboveground tree growth. In particular, although soil N may be strongly associated with SOC, such statistical coupling does not in itself demonstrate that N causally drives SOC accumulation. A key question is therefore whether total nitrogen (TN) emerges as the soil nutrient variable most strongly associated with SOC when soil moisture, phosphorus, bulk density, and plant traits are considered simultaneously.

The Qilian Mountains form an important ecological barrier in arid northwestern China, where *Picea crassifolia* is a dominant and ecologically important conifer. Previous studies have documented variation in soil C, N, and P characteristics of *Picea crassifolia* forests in relation to stand structure and environmental gradients (Liu et al., 2021; Feng et al., 2023; Liu et al., 2023). Recent regional evidence further shows that the distribution and population density of *Picea crassifolia* are jointly associated with topography, hydrothermal conditions, and soil depth, emphasizing the strong environmental dependence of this forest ecosystem (Wu et al., 2026a). Such environmental heterogeneity is important when interpreting apparent density effects because soil and topographic conditions may influence both stand development and belowground carbon processes. Nevertheless, comparatively little is known about whether aboveground tree growth and belowground SOC stocks exhibit concordant or contrasting patterns across stand-density conditions, especially when deeper soil layers are considered.

The novelty of this study lies in jointly evaluating aboveground tree growth, plant nutrient traits, and soil C, N, and P characteristics across the 0–80 cm soil profile under contrasting stand-density conditions. We focused on whether density conditions associated with greater tree growth were also associated with greater SOC and nutrient densities, and on identifying the soil and plant variables most strongly related to SOC. We hypothesized that: (1) tree growth and soil properties would differ among stand-density conditions; (2) density conditions favorable for tree growth would not necessarily coincide with those associated with greater SOC and nutrient densities; (3) SOC would be positively associated with soil water availability, nutrient status, and selected plant traits; and (4) soil total nitrogen would emerge as the strongest statistical correlate of SOC among the measured soil nutrient variables. To evaluate these hypotheses, we compared tree growth, leaf and root C, N, and P concentrations, soil physical properties, SOC, TN and TP contents and densities, and soil C:N:P stoichiometry across density conditions, supplemented by correlation analysis, redundancy analysis, and structural equation modeling. These multivariate analyses were interpreted as exploratory assessments of statistical associations rather than tests of definitive causal mechanisms.

## 2 Materials and methods

### 2.1 Study area

The study was conducted in a *Picea crassifolia* plantation in Minle County, Zhangye City, Gansu Province, northwestern China, in the eastern section of the Qilian Mountains (**Figure 1**). The study area is located in the shallow mountainous zone of the Qilian Mountains and is characterized by relatively gentle terrain. According to the characteristics of the selected stands, elevations and slopes varied within relatively narrow ranges (**Table 1**). The region has a temperate continental semi-arid alpine climate, characterized by low temperature, limited precipitation, strong evaporation, and pronounced seasonal water limitation. The mean annual temperature is approximately 3.2 °C, the frost-free period is approximately 118 days, and the annual sunshine duration is about 2,966.3 h. Mean annual precipitation is approximately 350 mm, whereas annual evaporation reaches approximately 1,638 mm. Climatic data were obtained from the Minle County Meteorological Station. The location of the study area and the distribution of the sampling stands are shown in **Figure 1**. The monthly mean temperature and precipitation in the study area from 2023 to 2025 are presented in **Figure 2**. The soils of the study area are classified as Haplic Cambisols. Before afforestation, the area had been affected by historical grazing and limited agricultural activities and was characterized by relatively sparse vegetation and a lack of an established tree layer. Herbaceous vegetation constituted the dominant pre-afforestation plant community, with *Agropyron cristatum* (L.) Gaertn. representing a common herbaceous species and *Caragana korshinskii* Kom. occurring as a common shrub in the surrounding area. These environmental conditions are representative of the relatively open and water-limited habitats of the low-elevation eastern Qilian Mountains (Shangguan et al., 2025).

**Figure 1:**
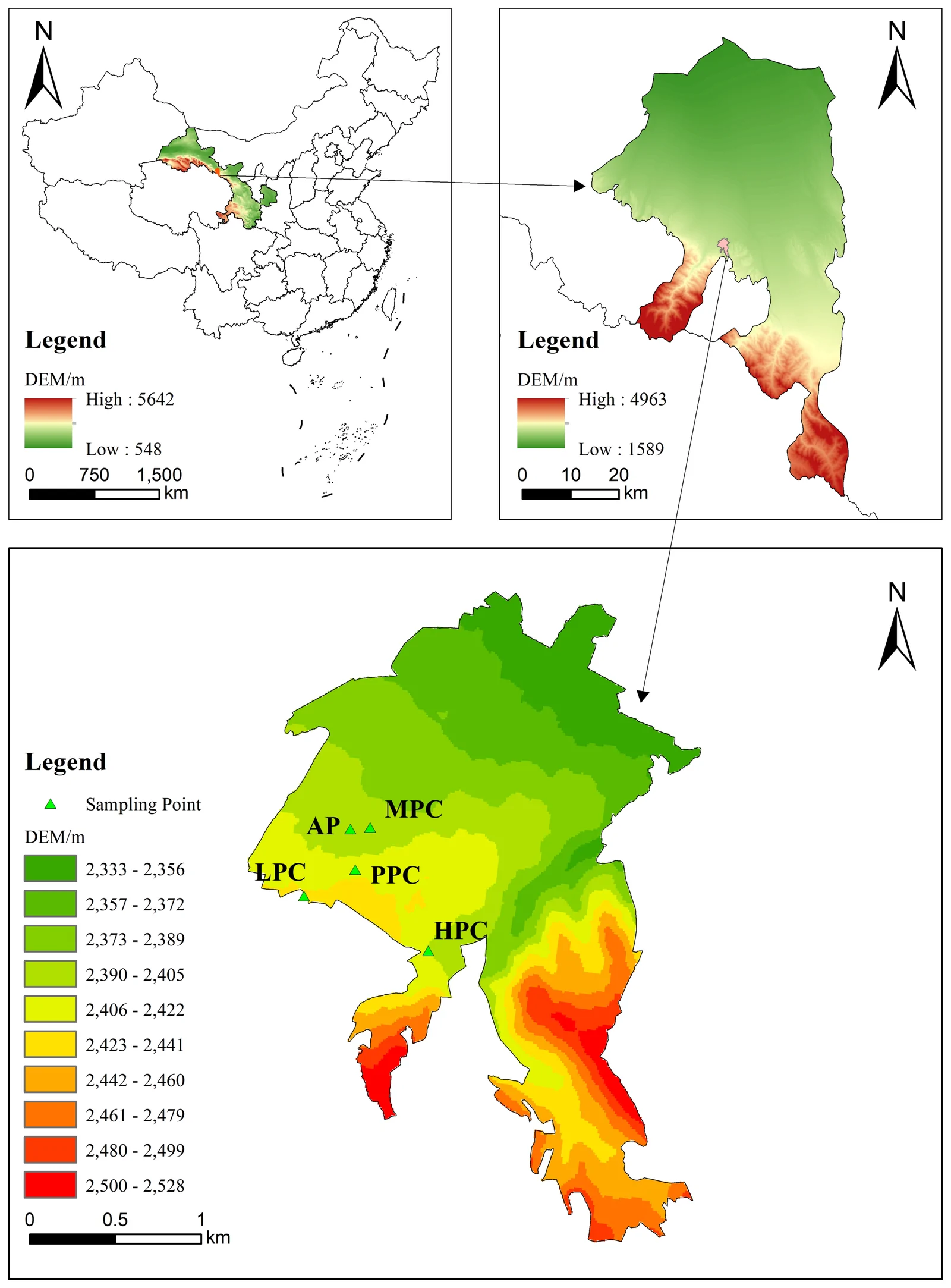
Geographical location of the research area, drawing review number GS (2024) 0650.

**Table 1:** Overview of sample sites.

Plot Name	Forest type	Latitude and Longitude	Altitude/m	Slope(°)	Spacing/m	planting density (stems ha^-^¹)
HPC	Pure forest	100.708849°E, 38.418981°N	2456.3	5	1.5×1.5	4444
LPC	Pure forest	100.700234°E, 38.421547°N	2476.6	10	2×2	2500
MPC	Pure forest	100.704530°E, 38.425227°N	2444.5	4	2×2.5	2000
AP	Pure forest	100.703195°E, 38.425101°N	2444.5	5	2×1.5	3333
PPC	Pure forest	100.703655°E, 38.423014°N	2464.2	8	1.5×2.5	2667

The labels HPC, LPC, MPC, AP, and PPC are arbitrary plot codes used solely to distinguish the five study stands and do not represent predefined density classes or other ecological categories.

**Figure 2:**
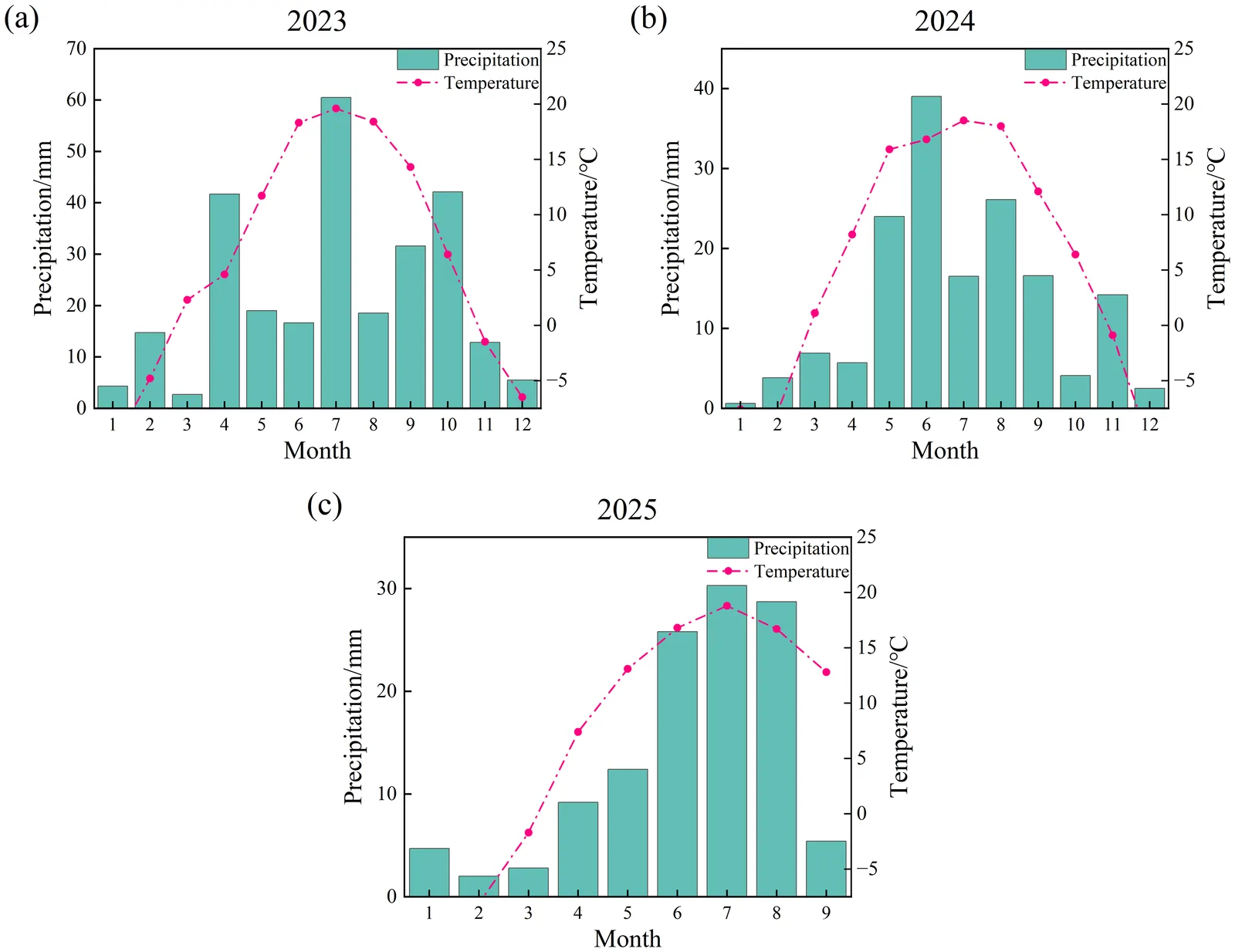
Monthly average temperature and precipitation in the study area (2023-2025).

Afforestation was initiated in May 2019 using high-quality *Picea crassifolia* seedlings obtained from the Longqu Seed Orchard in Zhangye, Gansu Province. All stands included in the present study were pure *Picea crassifolia* plantations established during the same period and under the same general regional climatic background. The stands differed primarily in planting spacing and nominal initial stand density. Five planting spacings were represented: 1.5 × 1.5, 2.0 × 2.0, 2.0 × 2.5, 2.0 × 1.5, and 1.5 × 2.5 m, corresponding to nominal initial densities of approximately 4,444, 2,500, 2,000, 3,333, and 2,667 stems ha^-^¹, respectively. HPC, LPC, MPC, AP, and PPC were treatment codes, their corresponding planting spacings, nominal initial densities, and actual stand densities are provided in **Table 1**.

### 2.2 Experimental design and plot selection

Field sampling was conducted in September 2025. Five stand-density treatments (HPC, LPC, MPC, AP, and PPC) were investigated. Each treatment comprised three spatially independent replicate stands, resulting in a total of 15 stands. Within each replicate stand, three 50 m × 50 m quadrats were established, with approximately 100 m between adjacent quadrats. Therefore, each treatment contained three independent replicate stands and nine quadrat-level observations. The replicate stands represented the independent stand-level replicates, whereas the three quadrats within each stand represented nested quadrat-level observational units.Replicate stands were selected according to the following criteria: (1) all stands were pure *Picea crassifolia* plantations established during the same afforestation period; (2) seedlings originated from the same regional seedling source and were of generally similar initial size; (3) replicate stands within each treatment had the same planting spacing and broadly similar stand structure; (4) stands occurred under comparable regional climatic and topographic conditions; and (5) stands showing obvious recent anthropogenic disturbance, severe grazing, fire, or large-scale pest or disease damage were excluded.

### 2.3 Sample collection and determination

#### 2.3.1 Tree survey and leaf sampling

All *Picea crassifolia* trees within each 50 m × 50 m quadrat were inventoried. Trees showing normal growth, intact crowns, no obvious stem damage, and no severe symptoms of disease or insect infestation were considered eligible for sampling. From these individuals, nine representative trees were selected from each quadrat based on ground diameter, tree height, and crown dimensions to represent the overall growth characteristics of the stand. Tree height was measured using an ultrasonic hypsometer (Vertex IV), ground diameter (GD) was measured using a vernier caliper, and crown length and crown width were measured using a handheld measuring pole. For each representative tree, healthy and mature leaves were collected using a pole pruner from the eastern, southern, western, and northern aspects of the crown and from the upper, middle, and lower canopy layers. Leaves collected from the same tree were thoroughly mixed to form one composite leaf sample per tree. The samples were placed in labeled envelopes and transported to the laboratory. In the laboratory, leaf samples were heated at 105 °C for 30 min and subsequently oven-dried at 65 °C to constant mass (Tong et al., 2021). The dried samples were ground using a ball mill and passed through a 100-mesh sieve for subsequent determination of plant nutrient concentrations. For tree growth and leaf nutrient traits, measurements from the nine representative trees within each quadrat were summarized to obtain one quadrat-level observation. Thus, nine quadrat-level observations were obtained for each treatment from the three replicate stands.

#### 2.3.2 Root sampling and preparation

Root samples were collected from the same representative trees used for leaf sampling. Surface soil near the stem was carefully removed, and the root system was traced until a main root directly connected to the target *Picea crassifolia* individual was identified. An approximately 15-cm-long segment of the main root was collected from each tree. Only clearly lignified coarse structural roots were sampled to avoid inclusion of fine roots. Adhering soil was gently removed in the field, and samples from individual trees were placed separately in labeled polyethylene bags and transported to the laboratory. Root samples were gently washed with tap water to remove adhering soil particles, rinsed with deionized water, and inspected to remove damaged, decayed, or visibly contaminated tissues. The cleaned root samples were heated at 105 °C for 30 min, oven-dried at 65 °C to constant mass, ground, and passed through a 100-mesh sieve for subsequent determination of C, N, and P concentrations (Li et al., 2022). Root nutrient measurements from the nine representative trees within each quadrat were summarized to obtain one quadrat-level observation, resulting in nine quadrat-level observations per treatment.

#### 2.3.3 Soil sampling

To minimize the immediate influence of the stem base while ensuring that samples were collected within the rooting zone of *Picea crassifolia*, soil sampling was conducted approximately 1 m horizontally from the nearest representative tree. After removing surface litter, soil samples were collected using a soil auger with an internal diameter of 3.5 cm from four soil layers: 0–20, 20–40, 40–60, and 60–80 cm. At each soil depth, three soil cores were collected within each quadrat and thoroughly composited to obtain one representative soil sample per soil layer per quadrat. At each soil depth, the three soil cores collected within a quadrat were composited to form one quadrat-level soil sample. Consequently, nine quadrat-level composite soil samples were obtained per treatment for each soil depth (three replicate stands × three quadrats per stand). The composite soil samples were placed in labeled self-sealing bags and transported to the laboratory. Undisturbed soil samples were collected separately from each of the four soil layers using a 100 cm³ cutting ring for determination of soil bulk density. In the laboratory, visible roots, stones, and other plant residues were removed. The samples were then thoroughly mixed, air-dried, ground, and passed through a 0.25-mm sieve for subsequent determination of soil C, N, and P concentrations.

#### 2.3.4 Determination of C, N, and P concentrations in leaves and roots

Leaf and root carbon concentrations (g·kg^-^¹) were determined using the potassium dichromate external heating method. Leaf and root nitrogen concentrations (g·kg^-^¹) were measured by the Kjeldahl method. For determination of plant total phosphorus, dried and ground leaf and root samples were digested with H_2_SO_4_–H_2_O_2_, and phosphorus concentration was determined colorimetrically using the molybdenum–antimony colorimetric method. Standard phosphorus solutions were used to construct the calibration curve, and reagent blanks and replicate samples were included for quality control. Plant phosphorus concentrations were expressed as g kg^-^¹ (Ouyang et al., 2025).

#### 2.3.5 Soil physical analysis

Soil gravimetric water content (SWC) was determined using the oven-drying method and calculated as follows (Wang and Li, 2025):


$$SWC(\%)=\frac{m_{1}-m_{2}}{m_{2}-m_{0}}\times 100$$

where m_0_ is the mass of the empty container (g), m_1_ is the mass of the container plus fresh soil (g), and m_2_ is the mass of the container plus oven-dried soil (g).

Bulk density (BD) was determined by using cutting ring method. Calculation formula for soil bulk density (Xiang et al., 2024):


$$BD(d)=({\text{W}}_{1}-{\text{W}}_{0})\times (1-\text{W}\%)/\text{V}$$

In the equation, the soil bulk density (g·cm^-^³) is denoted by d, the mass of the cutting ring (g) is represented by W_0_, the mass of the cutting ring plus fresh soil (g) is indicated by W_1_, the soil water content (%) is given by W%, and the volume of the cutting ring (100 cm³) is expressed as V.

#### 2.3.6 Soil nutrient analysis

Total nitrogen (TN, g·kg^-^¹) was determined by the semi-micro Kjeldahl method. Total phosphorus (TP, g·kg^-^¹) was measured by H_2_SO_4_ digestion followed by molybdenum-antimony anti-colorimetric analysis. Soil organic carbon (SOC, g·kg^-^¹) was determined using the potassium dichromate oxidation–external heating method (Xiang et al., 2024; Wang and Li, 2025).

The soil organic carbon density (SOC_Di_) is calculated using the following equation:


$$SOC_{Di}=\frac{SOC_{ci}\times BD_{i}\times H_{i}}{100}$$

where SOC_Di_ is the soil organic carbon density of the i-th layer (kg·m^-^²). SOC_Ci_ is the soil organic carbon content of the i-th layer (g·kg^-^¹). BD_i_ is the bulk density of the i-th layer (g·cm^-^³). Gravel is essentially absent in the bulk density samples, so it is not considered as a correction factor for organic carbon density estimation. H_i_ is the thickness of the i-th layer (cm). Soil total nitrogen density and total phosphorus density were calculated using the same equation as soil organic carbon density, with the corresponding TN and TP concentrations substituted for SOC concentration.

### 2.4 Statistical analysis

Data were organized using Microsoft Excel 2019. Each stand-density treatment comprised three spatially independent replicate stands, with three quadrats established within each stand. Accordingly, nine quadrat-level observations were obtained for each treatment, with the quadrats nested within the three replicate stands. Analysis of variance (ANOVA) was performed using IBM SPSS Statistics 26.0, and Duncan’s multiple range test was applied for multiple comparisons at *P* < 0.05. All measured values are expressed as means ± standard errors. Graphs were generated using Origin 2022.Pearson correlation analysis was conducted to examine relationships among leaf and root C, N, and P concentrations and soil physicochemical properties. Redundancy analysis (RDA) was performed in Canoco 5.0 to explore the relationships between tree characteristics, plant nutrient traits, and soil physicochemical properties, and the corresponding ordination plots were generated. Structural equation modeling (SEM) was performed using IBM SPSS Amos 26 Graphics to explore statistical associations and potential pathways among soil properties, plant traits, tree growth, and SOC. Correlation analysis, RDA, and SEM were treated as exploratory analyses.

## 3 Results

### 3.1 Tree growth and leaf–root C, N, and P concentrations across stand-density conditions

Tree growth characteristics differed significantly among the five stand-density conditions (*P* < 0.05) (**Table 2**). Tree height was significantly greater in the MPC and PPC treatments than in the HPC, LPC, and AP treatments. Ground diameter (GD) was highest in the PPC treatment and lowest in the HPC treatment, while no significant difference was observed between the LPC and MPC treatments. Crown length and crown width showed patterns similar to tree height, with significantly greater values in the MPC and PPC treatments than in the other three treatments. Overall, MPC and PPC were associated with greater aboveground tree growth than the other stand-density conditions.

**Table 2:** Tree characteristics of *Picea crassifolia* plantations under different densities.

Plot Name	Average Height/m	Average Ground Diameter (GD)/mm	Average Crown Length/m	Average Crown Width/m
HPC	1.10 ± 0.04 b	23.35 ± 0.72 d	0.75 ± 0.02 b	0.69 ± 0.02 b
LPC	1.14 ± 0.04 b	46.38 ± 1.63 b	0.87 ± 0.03 b	0.79 ± 0.02 b
MPC	1.61 ± 0.06 a	48.25 ± 2.12 b	1.03 ± 0.07 a	0.97 ± 0.07 a
AP	1.23 ± 0.04 b	38.74 ± 1.43 c	0.75 ± 0.05 b	0.69 ± 0.04 b
PPC	1.63 ± 0.07 a	55.20 ± 1.74 a	1.06 ± 0.05 a	0.97 ± 0.05 a

The data are the average values of repetitions. According to Duncan’s test analysis, different lowercase letters in the column indicate significant differences between different treatments (*P* < 0.05).

Leaf and root C, N, and P concentrations also differed significantly among stand-density conditions (*P* < 0.05) (**Figure 3**). Leaf C concentration was highest in HPC and lowest in MPC, whereas the remaining treatments showed intermediate levels. Leaf N concentration was higher in AP and PPC and lower in LPC, while leaf P concentration was highest in HPC and lowest in LPC. Root C concentration was higher in LPC and MPC and lower in HPC. Root N concentration was highest in AP and lowest in HPC and PPC, whereas root P concentration was highest in AP and lowest in PPC. Overall, the stand-density conditions associated with greater aboveground growth did not consistently correspond to those associated with higher C, N, and P concentrations in leaves and roots.

**Figure 3:**
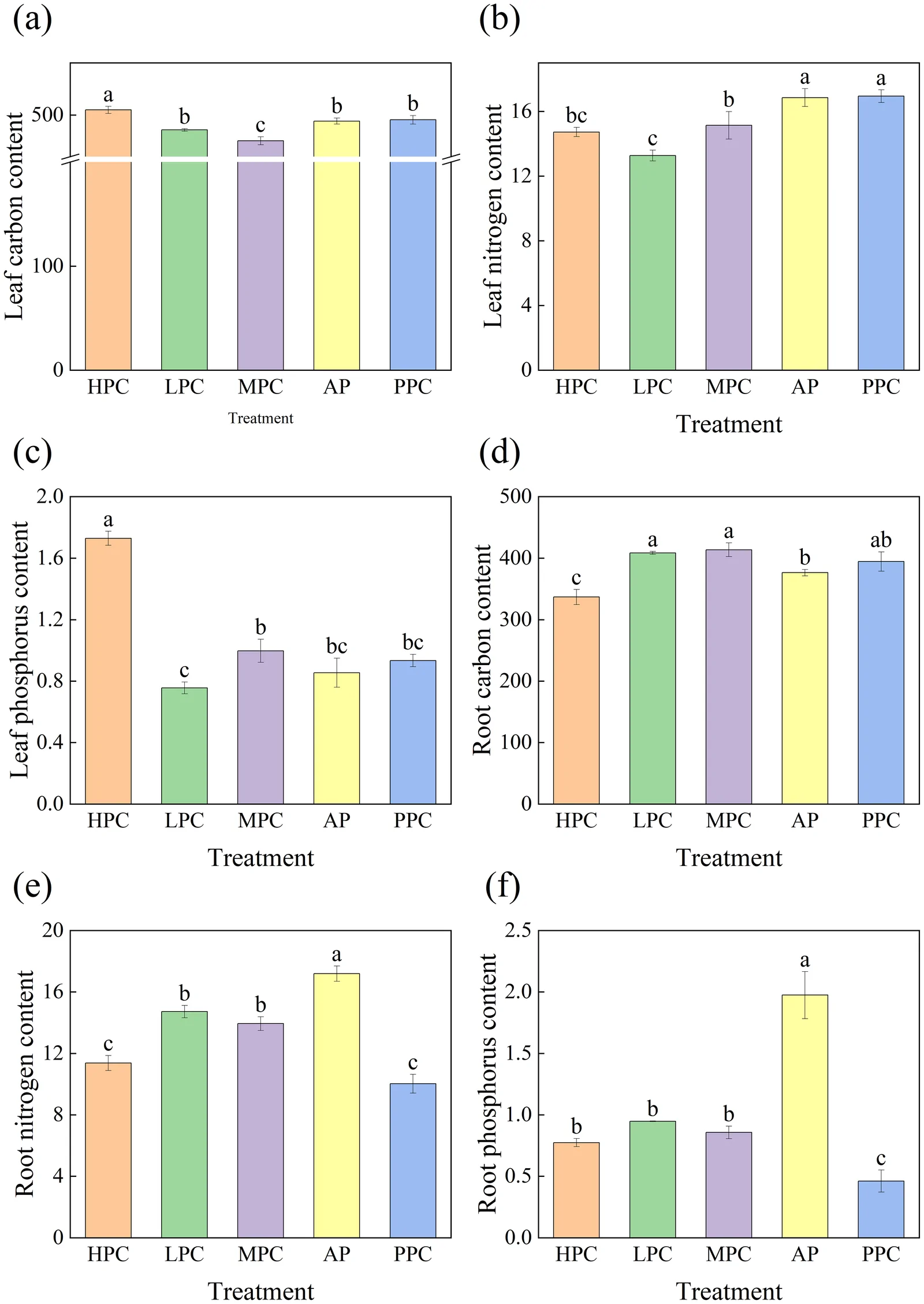
Leaf and root C, N, and P concentrations across stand-density treatments in the *Picea crassifolia* plantation. **(A)** Leaf C, **(B)** leaf N, **(C)** leaf P, **(D)** root C, **(E)** root N, and **(F)** root P concentrations. Values are means ± SE based on nine quadrat-level observations per treatment, with three quadrats nested within each of three independent replicate stands. HPC, LPC, MPC, AP, and PPC correspond to nominal initial planting densities of 4,444, 2,500, 2,000, 3,333, and 2,667 stems ha^-^¹, respectively. Different lowercase letters indicate significant differences among treatments at *P* < 0.05 based on the revised multiple-comparison procedure.

### 3.2 Soil physical properties across stand-density conditions

**Figure 4** shows variations in soil bulk density and soil water content across four soil depths among the five stand-density conditions. In the 0–20 cm soil layer, soil bulk density was significantly higher in the MPC treatment than in the other treatments, and it was significantly higher in the HPC and AP treatments than in the LPC and PPC treatments (*P* < 0.05). Soil water content in the 0–20 cm layer was significantly higher in the AP treatment than in the other treatments (*P* < 0.05). In the 20–40 cm layer, both soil bulk density and soil water content were significantly higher in the AP treatment than in the other treatments (*P* < 0.05). Soil bulk density was significantly higher in the HPC and LPC treatments than in the MPC and PPC treatments, while soil water content was significantly higher in the MPC treatment than in the HPC, LPC, and PPC treatments (*P* < 0.05). In the 40–60 cm layer, soil bulk density was significantly higher in the LPC, AP, and PPC treatments than in the HPC and MPC treatments, whereas soil water content was significantly higher in the HPC, MPC, and AP treatments than in the LPC and PPC treatments (*P* < 0.05). In the 60–80 cm layer, soil bulk density was significantly higher in the PPC treatment than in the other treatments, and soil water content was significantly higher in the AP treatment than in the other treatments. Soil bulk density in the MPC and AP treatments was significantly higher than that in the HPC and LPC treatments. With respect to vertical variations among soil layers, soil bulk density in the HPC and AP treatments was significantly higher in the 0–20 cm and 20–40 cm layers than in the deeper layers (40–80 cm). In the LPC treatment, soil bulk density was significantly highest in the 20–40 cm layer, whereas in the MPC treatment it was significantly highest in the 0–20 cm layer. In the PPC treatment, soil bulk density was significantly lowest in the 20–40 cm layer. Soil water content in the HPC, LPC, MPC, and PPC treatments tended to increase with soil depth, and values in the 40–60 cm and 60–80 cm layers were significantly higher than those in the 0–20 cm and 20–40 cm layers. In the AP treatment, soil water content was significantly lowest in the 20–40 cm layer.

**Figure 4:**
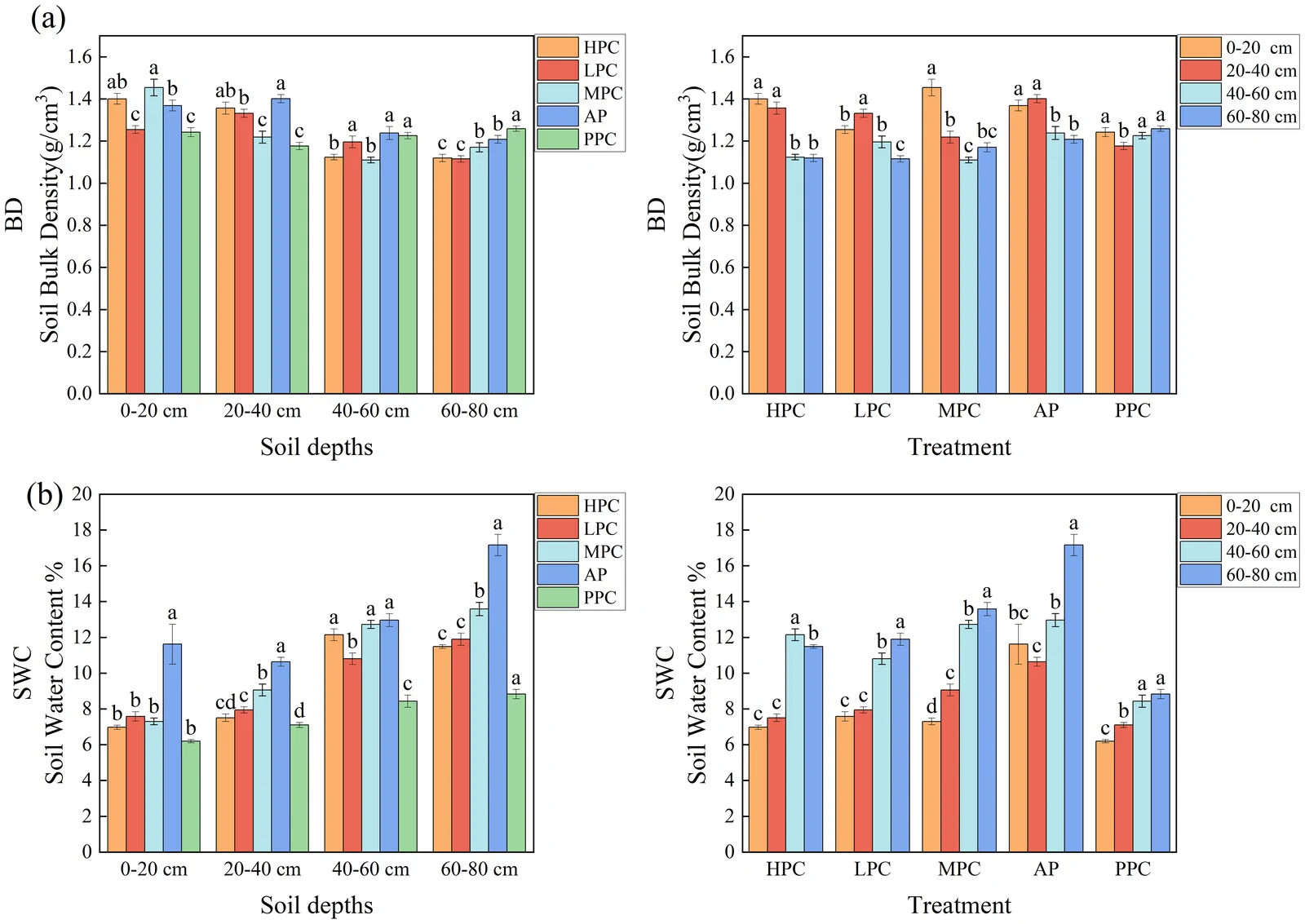
Soil bulk density (BD; g cm^-^³) and gravimetric soil water content (SWC; %) across stand-density treatments and soil depths. **(A)** BD; **(B)** SWC. Left panels compare treatments within each soil depth, whereas right panels compare soil depths within each treatment. Values are means ± SE. Three independent replicate stands were included per treatment, with three quadrats nested within each stand. Different lowercase letters indicate significant pairwise differences at *P* < 0.05.

### 3.3 Soil C, N, and P contents, density, and stoichiometric ratios across stand-density conditions

**Figure 5** shows the variation in SOC, TN, and TP contents across soil depths among the five stand-density conditions. In the 0–20 cm soil layer, SOC, TN, and TP contents were significantly higher in the AP treatment than in the other treatments, while no significant differences were observed among the HPC, LPC, and MPC treatments. In the 20–40 cm layer, SOC content was significantly highest in the HPC and MPC treatments. TN content was significantly higher in the MPC treatment than in the other treatments, and TP content was significantly highest in the HPC treatment. In the 40–60 cm layer, SOC, TN, and TP contents were significantly higher in the HPC, LPC, MPC, and AP treatments than in the PPC treatment. In the 60–80 cm layer, SOC content was significantly higher in the LPC treatment than in the other treatments. TN content was significantly higher in the AP treatment than in the other treatments, and TP content was significantly higher in the AP treatment than in the MPC and PPC treatments. Across all soil layers, the lowest SOC, TN, and TP contents were consistently recorded in the PPC treatment. SOC and TN exhibited generally consistent vertical patterns among the different stand-density conditions. In the HPC treatment, SOC and TN contents in the 40–60 cm layer were significantly higher than those in the other layers, following the order 40–60 cm > 60–80 cm > 0–20 cm > 20–40 cm. In the LPC and MPC treatments, SOC and TN contents in the 40–60 cm and 60–80 cm layers were significantly higher than those in the surface soil (0–20 cm). In the AP treatment, SOC and TN contents in the 20–40 cm layer were significantly lower than those in the other soil layers. In the PPC treatment, SOC and TN contents decreased significantly with increasing soil depth. For all treatments, TP content was significantly highest in the surface soil (0–20 cm), and no significant differences were detected among the deeper soil layers.

**Figure 5:**
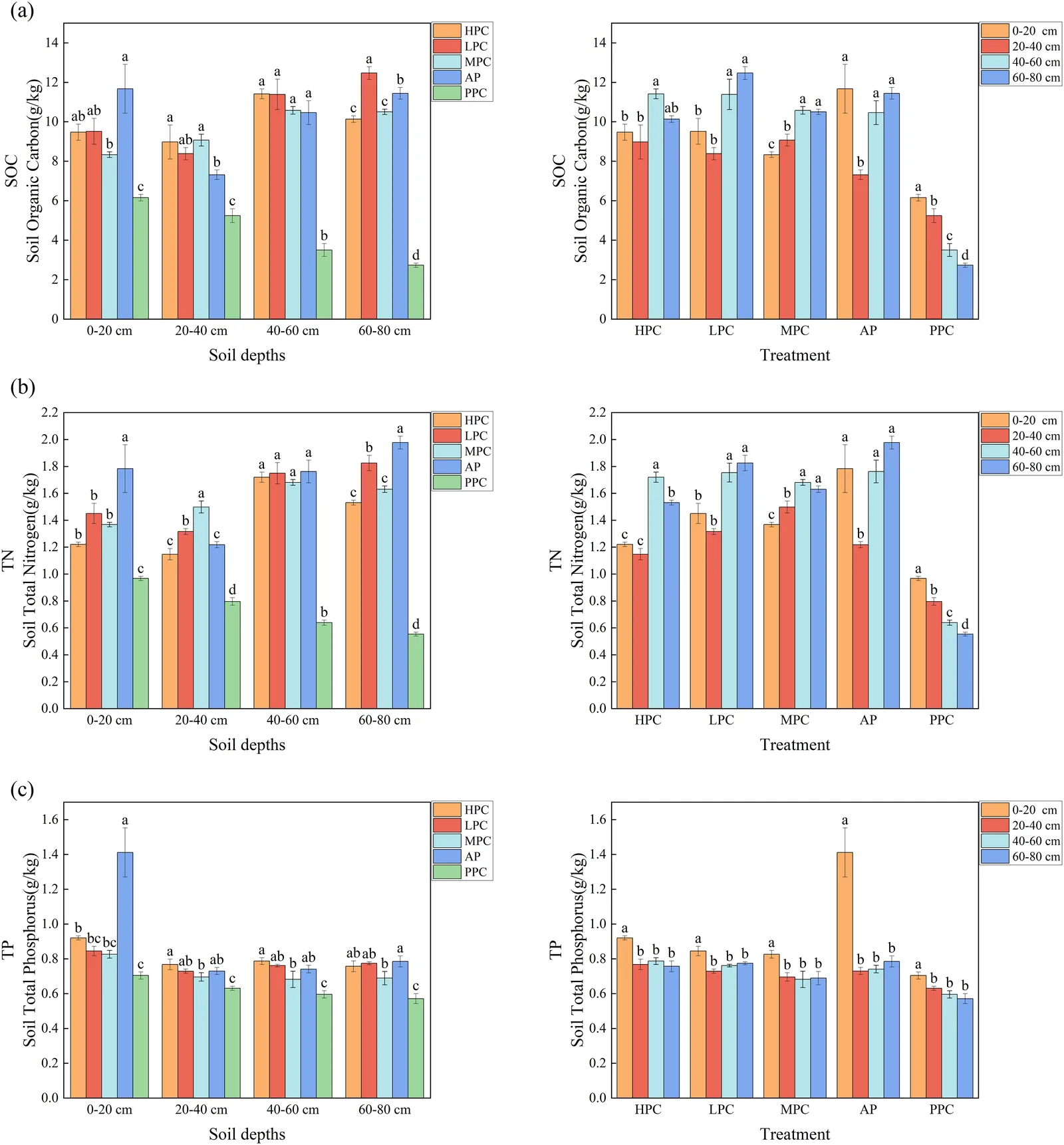
Soil organic carbon (SOC), total nitrogen (TN), and total phosphorus (TP) concentrations (g kg^-^¹) across stand-density treatments and soil depths. **(A)** SOC, **(B)** TN, and **(C)** TP. Left panels compare treatments within soil depths, whereas right panels compare soil depths within treatments.

**Figure 6** illustrates the variations in soil organic carbon density (SOC density), total nitrogen density (TN density), and total phosphorus density (TP density) in different soil layers among the five stand-density conditions. In the 0–20 cm soil layer, SOC, TN, and TP densities were significantly higher in the AP treatment than in the other treatments, while no significant differences were observed among the HPC, LPC, and MPC treatments. In the 20–40 cm layer, SOC density was significantly highest in the HPC treatment, TN density was significantly highest in the MPC treatment, and TP density was significantly highest in the HPC, LPC, and AP treatments. In the 40–60 cm and 60–80 cm layers, SOC, TN, and TP densities in the LPC and AP treatments were higher than those in the other treatments. The PPC treatment exhibited significantly lower SOC, TN, and TP densities in all soil layers compared to the other treatments. Regarding vertical distribution within each treatment, TN density in the HPC treatment was significantly higher in the 40–60 cm layer than in the remaining soil layers. In the LPC treatment, SOC and TN densities in the 40–60 cm and 60–80 cm layers were significantly higher than those in the surface soil, and values in the 0–20 cm layer were higher than those in the 20–40 cm layer. In the MPC treatment, relatively higher SOC and TN densities were recorded in the 0–20 cm and 60–80 cm layers, whereas no significant differences were detected between the 20–40 cm and 40–60 cm layers. In the AP treatment, the highest SOC and TN densities were found in the surface 0–20 cm layer, and in the 20–80 cm layers, SOC and TN densities increased significantly with increasing soil depth. In the PPC treatment, SOC and TN densities decreased significantly with increasing soil depth. For all treatments, TP density in the surface soil (0–20 cm) was significantly higher than that in the deeper soil layers.

**Figure 6:**
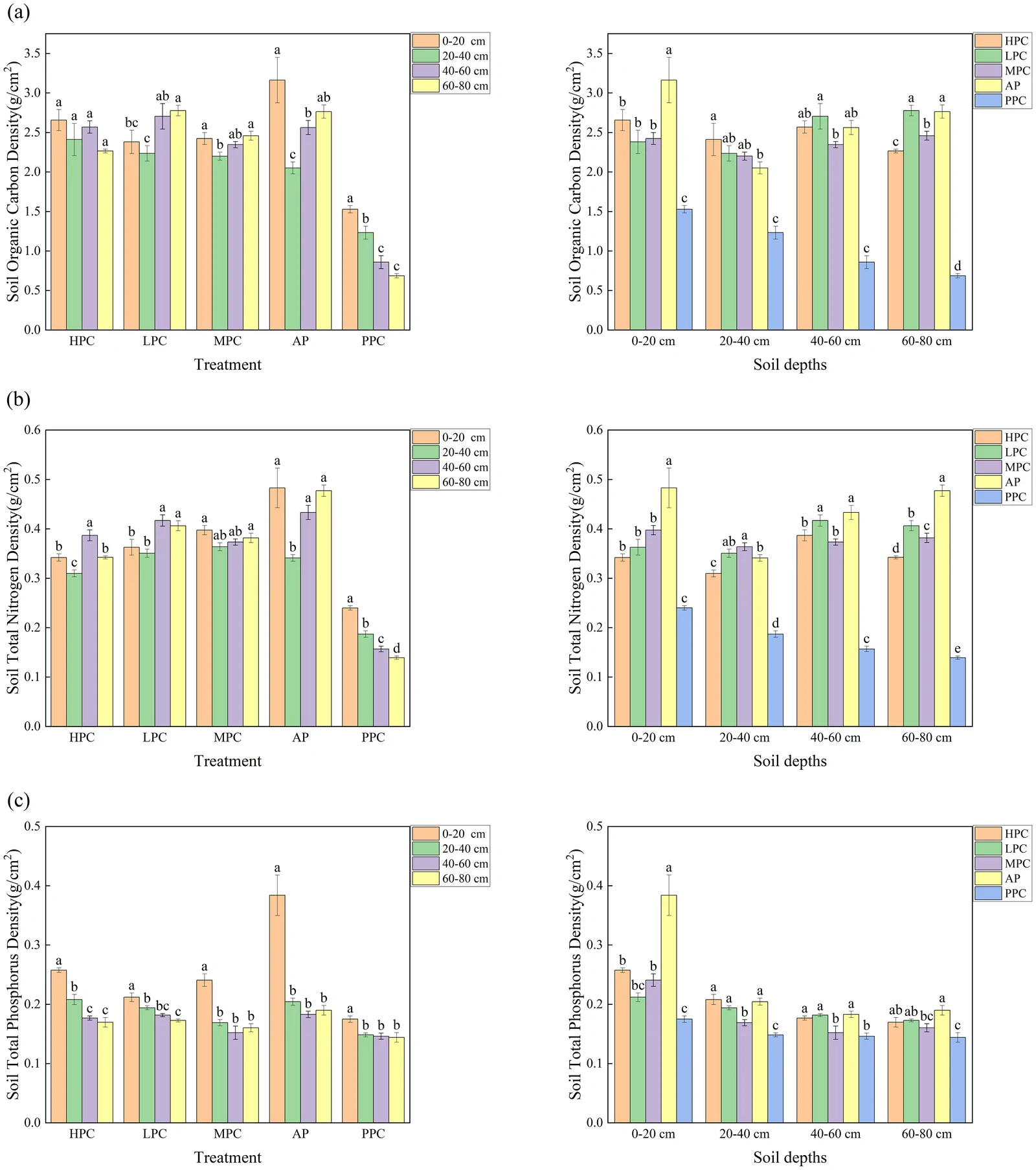
Soil carbon, nitrogen, and phosphorus storage in artificial forests of *Picea crassifolia* with different densities. In the figure, SOCD represents soil organic carbon density, TND represents soil total nitrogen density, and TPD represents soil total phosphorus density. The same below.

**Figure 7** illustrates the variations in soil C:N, C:P, and N:P ratios in different soil layers among the five stand-density conditions. In the 0–20 cm layer, the C:N ratio was significantly higher in the HPC treatment than in the other treatments, the C:P ratio was significantly highest in the LPC treatment, and the N:P ratio was significantly higher in the LPC and MPC treatments than in the other treatments (*P* < 0.05).In the 20–40 cm layer, the C:N ratio was significantly higher in the HPC treatment than in the other treatments, and the C:P and N:P ratios were significantly higher in the MPC treatment than in the other treatments (*P* < 0.05).In the 40–60 cm layer, the C:N ratio was significantly higher in the HPC and LPC treatments than in the PPC treatment. The C:P and N:P ratios were significantly higher in the HPC, LPC, MPC, and AP treatments than in the PPC treatment, with the highest values recorded in the MPC treatment. In the 60–80 cm layer, the C:N ratio was significantly higher in the HPC, LPC, and MPC treatments than in the AP and PPC treatments. The N:P ratio differed among HPC, LPC, MPC, and AP, with AP showing the highest value. With respect to vertical changes within each treatment, in the HPC treatment the surface soil C:N ratio was significantly higher than that in the deeper layers, whereas the C:P and N:P ratios initially increased and then decreased with increasing soil depth. No significant differences in the C:N ratio were detected among soil layers in the LPC and MPC treatments. In the AP treatment, the C:N ratio was significantly higher in the 0–20 cm layer than in the other layers. In the LPC, MPC, and AP treatments, the C:P and N:P ratios were significantly higher in the deeper soil layers than in the surface soil. In the PPC treatment, the C:N, C:P, and N:P ratios in the surface 0–40 cm layers were significantly higher than those in the deeper layers.

**Figure 7:**
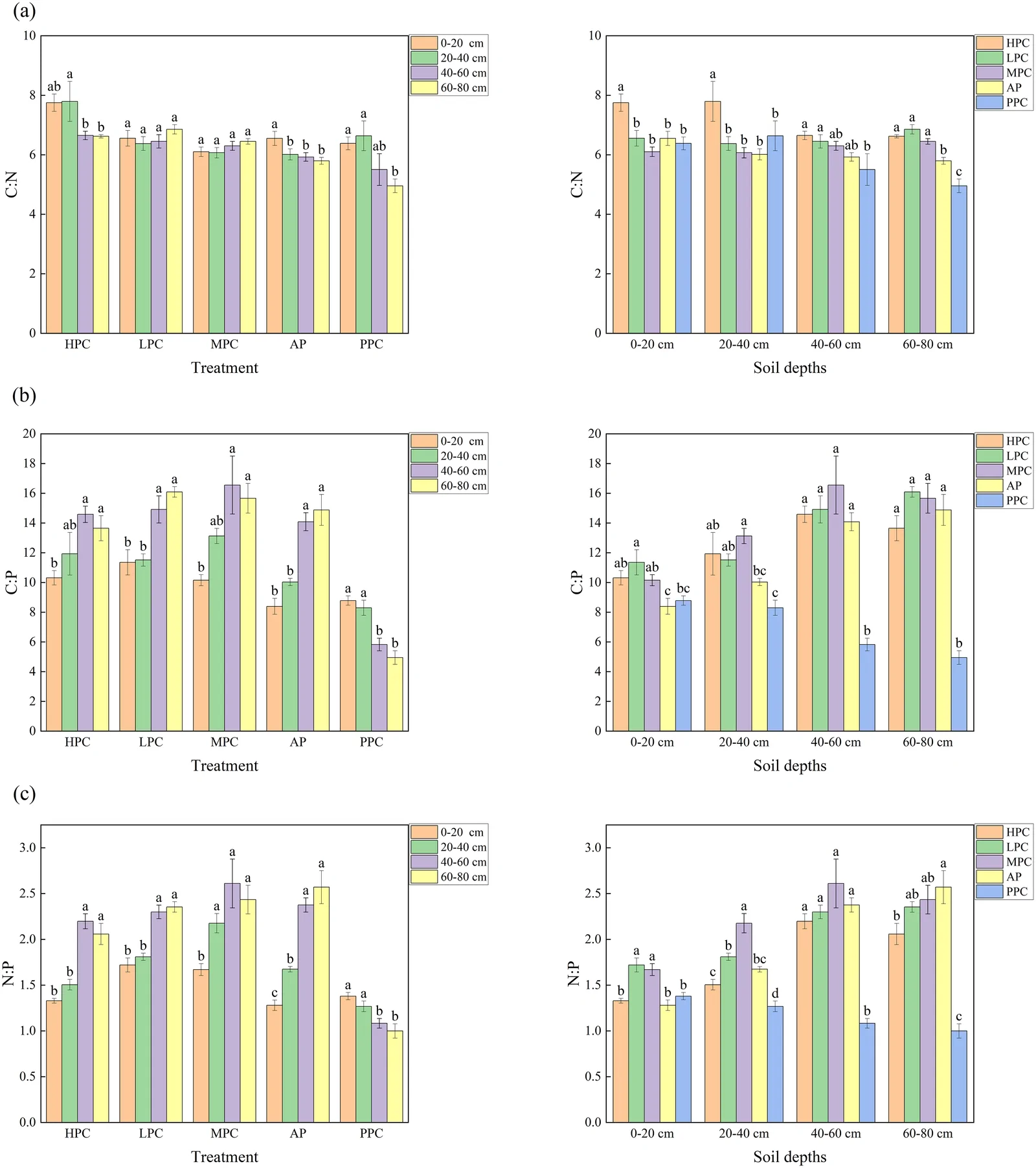
Soil carbon, nitrogen, and phosphorus stoichiometry ratios of different densities of *Picea crassifolia* plantations.

### 3.4 Correlations among soil physicochemical properties across stand-density conditions

Correlation analysis of soil properties in different soil layers is shown in **Figure 8**. In the 0–20 cm layer, significant positive correlations were observed among soil water content, soil organic carbon (SOC), total nitrogen (TN), total phosphorus (TP), SOC density, and TN density (*P* < 0.05). Soil bulk density, soil water content, TP, and TP density were negatively correlated with soil C:P and N:P ratios, whereas soil bulk density was significantly positively correlated with SOC density, TN density, and TP density (*P* < 0.05). In the 20–40 cm layer, positive correlations were found among soil bulk density, soil water content, SOC, TN, TP, and SOC density. Soil water content, SOC, TN, SOC density, and TN density exhibited significant positive correlations with soil N:P ratio (*P* < 0.05), while soil water content, TN, and TN density were negatively correlated with soil C:N ratio.In the 40–60 cm layer, significant positive correlations were detected among soil water content, SOC, TN, SOC density, TN density, and N:P ratio. Soil bulk density was significantly negatively correlated with soil water content, SOC, TN, C:N ratio, C:P ratio, and N:P ratio (*P* < 0.05). In the 60–80 cm layer, soil bulk density showed significant negative correlations with SOC, TN, TP, SOC density, TN density, C:N ratio, C:P ratio, and N:P ratio (*P* < 0.05). Positive correlations were observed among soil water content, SOC, TN, TP, and related indicators.

**Figure 8:**
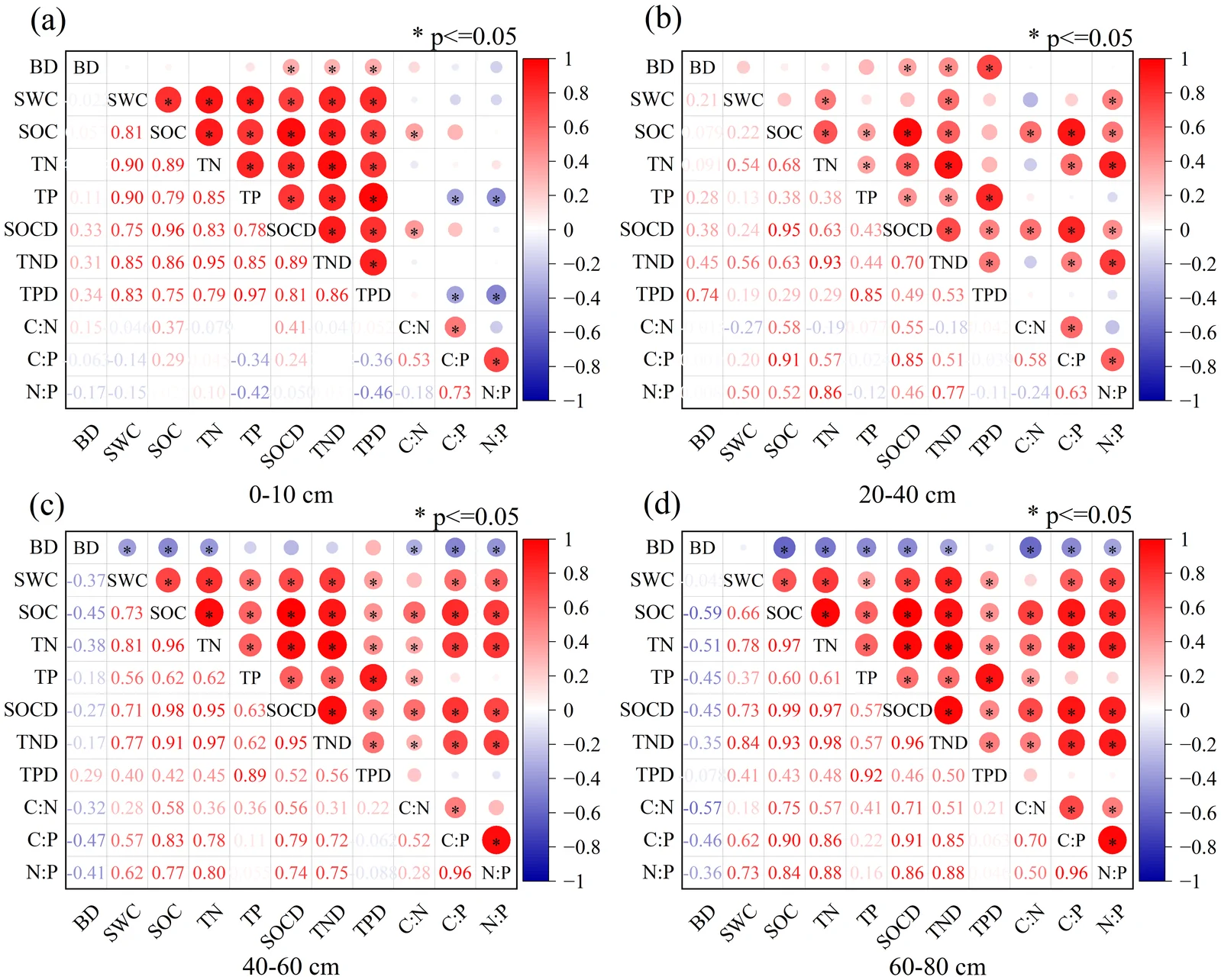
Correlation analysis of soil physicochemical properties in artificial forests of *Picea crassifolia* with different densities. The red color in the figure represents positive correlation, the blue color represents negative correlation, and * represents significant correlation between indicators (*P* < 0.05).

### 3.5 Redundancy analysis (RDA) of soil physicochemical properties and tree characteristics across stand-density conditions

The RDA ordination showed clear differentiation among the five stand-density conditions (**Figure 9**). Soil factors explained 46.19% of the total variation in tree characteristics, with Axis 1 and Axis 2 accounting for 36.21% and 9.98% of the variation, respectively. The contribution rates of TP, TN, SOC, soil water content, and bulk density were 59.9%, 20.4%, 8.0%, 7.4%, and 4.2%, respectively. Soil TP showed the highest contribution to the observed differentiation among the stand-density conditions. Soil water content, SOC, TN, and TP were negatively associated with GD, tree height, crown width, and leaf N concentration in the RDA ordination, but were positively associated with root P, root N, and leaf P concentrations. Positive correlations were observed among GD, tree height, crown width, leaf N content, and root carbon (C) content. The HPC and AP treatments were mainly distributed in the negative region of RDA1 and were significantly associated with leaf P content, leaf C content, root P, and soil TP. The LPC treatment was concentrated in the negative regions of both RDA1 and RDA2, with its sample points overlapping with soil water content, SOC, TN, and root N content, indicating greater soil nutrient accumulation under the LPC treatment. The PPC treatment was distributed in the positive region of RDA1, and its sample points overlapped with tree height, crown width, and GD, suggesting that this treatment promoted aboveground tree growth.

**Figure 9:**
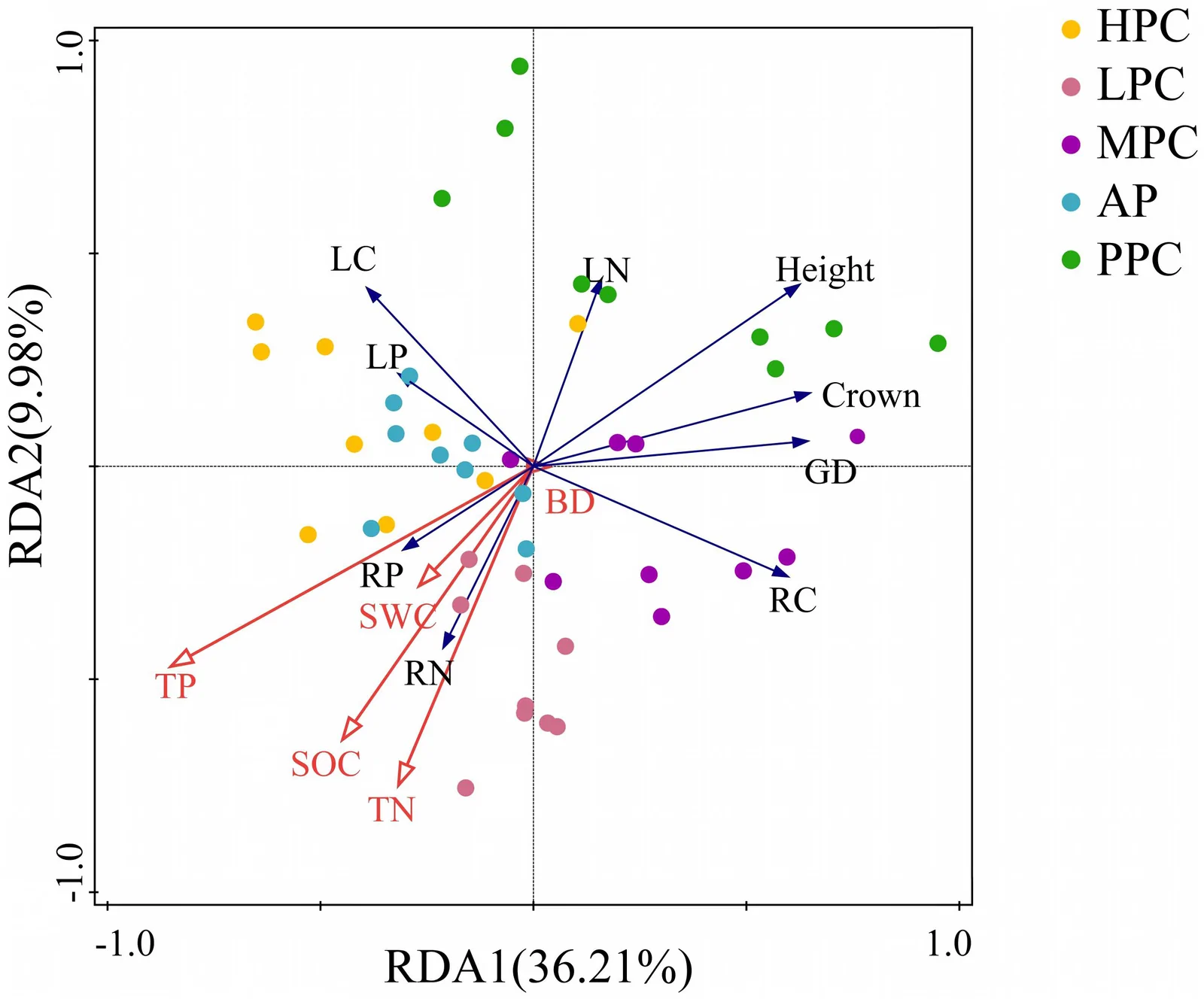
Redundancy analysis of soil physicochemical properties and tree characteristics in artificial forests of *Picea crassifolia* with different densities.

### 3.6 Exploratory structural equation modeling of factors associated with SOC

An exploratory structural equation model (SEM) was used to assess statistical associations among soil water content, soil nutrients, plant nutrient traits, tree growth, and SOC(**Figure 10**). Soil water content exhibited highly significant positive standardized path to soil total nitrogen (TN) and total phosphorus (TP) (*P* < 0.001), a positive effect on soil bulk density, and a highly significant negative effect on soil organic carbon (SOC). Soil water content was also indirectly associated with SOC through soil factors, plant nutrient traits, and tree growth variables. Soil TN showed significant positive standardized paths to leaf P, root C, and root N concentrations, and negative standardized paths to soil bulk density and leaf C concentration. Soil TP showed a highly significant positive standardized path to soil TN and highly significant negative standardized paths to tree height and root C concentration (P < 0.001). Leaf N, leaf C, and root N concentrations showed significant positive standardized paths to SOC, whereas root C concentration showed a negative standardized path to SOC. The SEM accounted for 92.5% of the variance in SOC in the analyzed dataset. The R^2^ values for TN, soil bulk density (BD), and TP were 0.730, 0.192, and 0.214, respectively, indicating that soil water content accounted for a substantial portion of the variance in these soil factors. For tree organ nutrients and growth indicators, the R^2^ values were 0.706 for root N, 0.425 for leaf P, 0.522 for tree height, and 0.657 for crown width, demonstrating that soil factors provided a reasonably good explanation of the variance in tree organs and growth. Furthermore, the standardized total effects derived from the SEM showed that soil TN had the strongest total effect on SOC (1.064), followed by soil water content and soil TP, all of which were positive. Leaf C, root N, and leaf P exerted weaker positive total effects on SOC, whereas tree height, soil bulk density, and root C exhibited negative total effects. Among the variables included in the SEM, soil TN showed the strongest statistical association with SOC, while soil water content, TP, and plant nutrient traits were also associated with SOC through direct or indirect model pathways.

**Figure 10:**
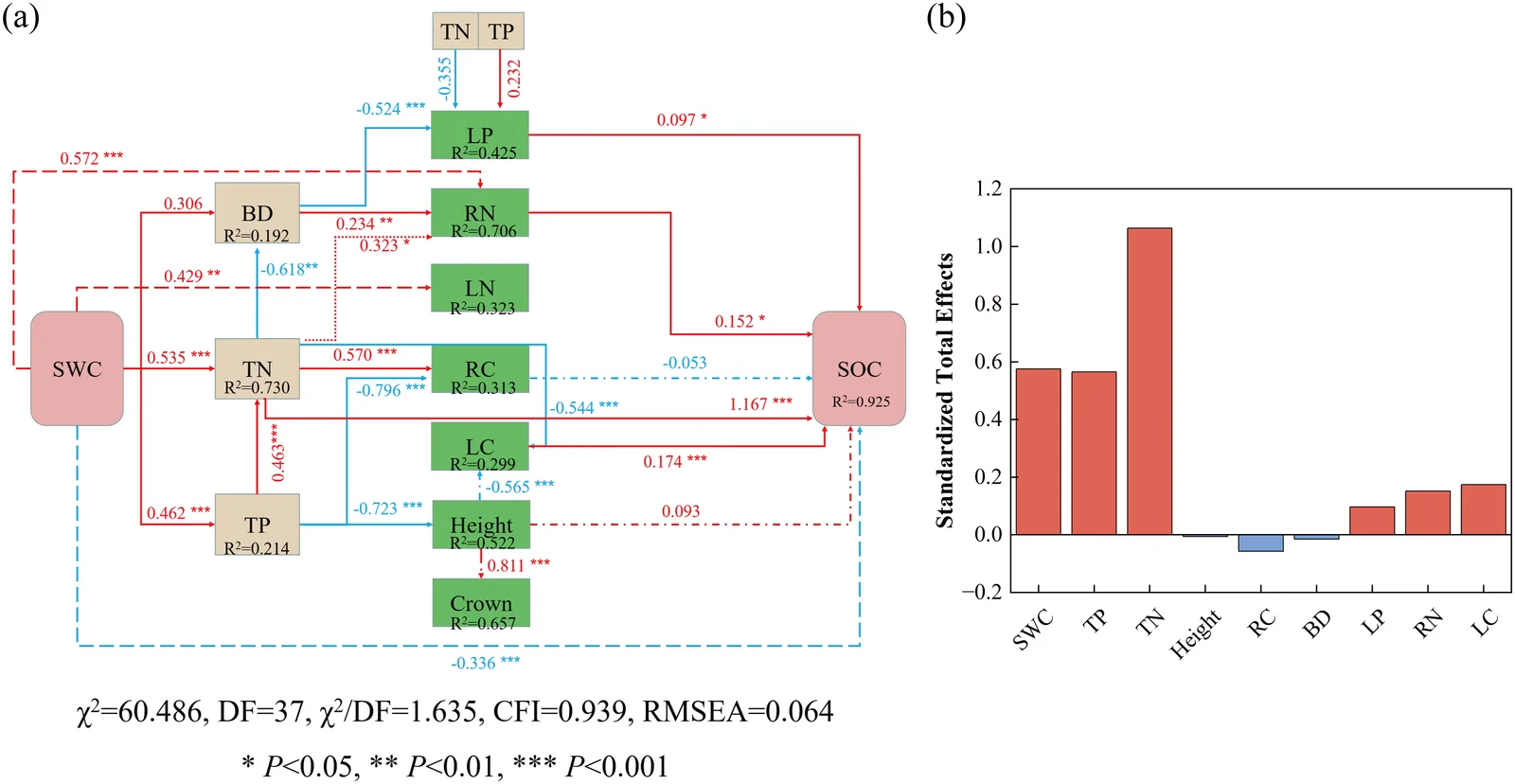
Structural equation model **(A)** and effect values **(B)** between tree characteristics and soil physicochemical properties.

## 4 Discussion

### 4.1 Contrasting responses of tree growth and soil carbon storage across stand-density conditions

The present study revealed a clear contrast between aboveground tree growth and belowground soil carbon and nutrient status across stand-density conditions. Tree height, ground diameter, crown length, and crown width were generally greater under the MPC and PPC treatments, whereas these growth-favorable conditions did not consistently correspond to higher soil C, N, and P levels. In particular, PPC was associated with relatively low SOC, TN, and TP contents and densities across the soil profile, whereas AP and LPC showed comparatively higher soil C and nutrient densities in several surface and deep soil layers. These results support our hypothesis that stand-density conditions favorable for individual tree growth do not necessarily coincide with those associated with greater soil carbon and nutrient storage. Thus, the response to stand density appears to involve a trade-off between aboveground growth and belowground resource storage rather than a single density condition optimizing both functions.

Such divergence is consistent with the broader view that density regulation alters competition for light, water, and nutrients and can therefore generate different responses at the individual-tree and ecosystem levels. Recent work in water-limited Larix principis-rupprechtii plantations showed that the density associated with an optimal balance between individual growth and stand-level carbon storage changes with stand development and moisture availability, highlighting the context dependence of growth–carbon trade-offs (Wu et al., 2026b). Similarly, stand-density regulation has been shown to modify labile SOC fractions, extracellular enzyme activities, and soil respiration, with soil moisture among the important covarying factors (Liu et al., 2025)). These findings help explain why greater tree growth does not necessarily imply greater soil C storage. Earlier work also illustrates the lack of a universal density response: higher stand density increased soil C storage in young larch plantations by increasing litter and root C inputs (Na et al., 2021), whereas density–carbon relationships can shift with water availability, stand age, and ecosystem context (Sterck et al., 2021; Wu et al., 2026a).

This interpretation is particularly relevant to the Qilian Mountains. A recent study of *Picea crassifolia* plantations in the region found substantial differences in carbon storage among planting configurations and emphasized the large contribution of soil, including deeper soil layers, to total ecosystem carbon storage (Shangguan et al., 2025). Although that study addressed planting patterns rather than the density conditions examined here, both studies indicate that aboveground performance alone is insufficient for evaluating plantation carbon function. Accordingly, our results should be interpreted as evidence of density-associated functional differentiation rather than evidence for a universally optimal stand density.

### 4.2 Depth dependent variation in soil water and C, N, and P characteristics

The effects associated with stand density were also strongly dependent on soil depth. We observed substantial vertical variation in soil water content, SOC, TN, TP, their densities, and C:N:P stoichiometric ratios. Soil water content generally increased toward deeper layers in several treatments, whereas the distributions of SOC and nutrients differed among stand-density conditions. AP showed comparatively favorable soil C and nutrient status in the surface layer, while LPC and AP generally maintained higher SOC, TN, and TP densities in parts of the 40–80 cm profile. These patterns indicate that evaluation of stand-density responses based only on surface soil may overlook important belowground differences. Recent plantation studies similarly demonstrate that soil depth can modify stand-structure effects on soil physical and chemical properties. In Cryptomeria japonica plantations, stand structure and soil depth jointly influenced bulk density, water-holding characteristics, SOC, TN, TP, and stoichiometric ratios (Luo et al., 2025). In semiarid black locust plantations, stand density was associated with differences in tree growth, soil nutrients, and the vertical behavior of soil water, with deeper soil water showing greater temporal stability (Zhai et al., 2024). These observations are relevant to the Qilian Mountains because water availability strongly constrains vegetation development in semiarid mountain environments. They also reinforce the need to consider the whole 0–80 cm profile rather than inferring belowground responses from surface soils alone.

Belowground plant inputs provide one plausible explanation for some of the observed depth-dependent patterns, but this interpretation requires caution. Experimental evidence from forest ecosystems has shown that root-derived C inputs can contribute more strongly than aboveground litter to the formation and stabilization of microbial necromass C (Jia et al., 2022). More recent work has also shown that deep-rooted perennial vegetation can alter microbial respiration and the chemical composition of SOC differently in surface and deep soils (Min et al., 2025). These studies provide a mechanistic basis for hypothesizing that variation in root distribution, turnover, and rhizosphere processes may contribute to the vertical patterns observed in our study.The vertical differences in C:N:P ratios further suggest that C, N, and P were not distributed proportionally across the soil profile. In particular, TP generally exhibited a stronger surface concentration than SOC and TN, while C:P and N:P ratios varied with both treatment and depth. Such patterns are consistent with the different biogeochemical controls on these elements: C and N are strongly linked to biological inputs and decomposition, whereas soil P can be more strongly constrained by mineral sources, adsorption, and redistribution. Nevertheless, the stoichiometric ratios measured here should not be interpreted directly as evidence of N or P limitation because available nutrient pools, microbial nutrient demand, and nutrient-acquisition processes were not directly quantified.

### 4.3 Soil nitrogen as the strongest statistical correlate of SOC

A major finding of this study was the close statistical coupling between SOC and soil N. Positive associations between SOC and TN occurred across several soil depths, and the exploratory SEM identified TN as the variable with the strongest total association with SOC among the measured soil factors. This result is consistent with our fourth hypothesis that TN would emerge as the strongest statistical correlate of SOC. Importantly, this finding should be interpreted as an association rather than evidence that TN causally drives SOC formation. The SEM describes the covariance structure among the measured soil and plant variables, and its pathways should therefore be viewed as hypotheses about potential relationships that require experimental validation. The strong SOC–TN association is biologically plausible because forest C and N cycles are closely coupled through plant production, litter and root inputs, microbial processing, and stabilization of organic matter. A large-scale forestation study across northern China demonstrated that soil N availability was closely linked to the relative allocation of carbon between vegetation and soil, and that increases in biomass C were not necessarily accompanied by parallel increases in SOC (Hong et al., 2023). Field manipulation in an alpine spruce forest further showed that N addition was associated with greater rhizosphere C sequestration and microbial-necromass formation than in bulk soil, illustrating how N availability can modify belowground C stabilization processes (Wang et al., 2023). These studies support the interpretation that N is an important component of SOC dynamics, while also emphasizing that the direction and magnitude of C–N coupling depend on ecosystem context.

The multivariate analyses also suggest that SOC variation cannot be understood from TN alone. RDA identified TP as the soil variable contributing most strongly to the multivariate differentiation among the measured tree and plant traits, whereas the SEM identified TN as the factor most strongly associated with SOC specifically. These findings are not contradictory because the two analyses address different statistical questions: RDA describes multivariate covariation among sets of variables, whereas the SEM focuses on the network of associations surrounding SOC. The model also linked soil water content, TN, TP, plant nutrient traits, and tree growth with SOC through multiple direct or indirect pathways. Recent density-gradient research has similarly shown that soil moisture, microbial biomass, SOC fractions, extracellular enzyme activities, and respiration covary under different plantation densities and ages (Liu et al., 2025). Thus, soil water availability may influence SOC through several coupled processes, but the present SEM should be interpreted as suggesting these pathways rather than demonstrating them experimentally.

### 4.4 Site-specific management implications and study limitations

Under the environmental and stand conditions examined in the present study, the contrasting responses of tree growth and soil carbon storage provide a useful basis for formulating site-specific density-management hypotheses. The MPC and PPC treatments were associated with greater tree height, ground diameter, and crown development, suggesting that these density conditions may be more favorable when individual-tree growth is a primary management objective. In contrast, the AP and LPC treatments generally showed greater SOC and nutrient storage in several soil layers, indicating their potential value when greater emphasis is placed on soil carbon retention and nutrient conservation. These contrasting responses suggest that density management in *Picea crassifolia* plantations may need to balance aboveground growth with belowground carbon and nutrient functions rather than pursue a single universally optimal density. Accordingly, the relative advantages of AP and LPC for soil carbon storage should be viewed as a site-specific management hypothesis that warrants further testing rather than as a definitive prescription.

Some considerations should be taken into account when interpreting these implications. The present study focused on *Picea crassifolia* plantations established during the same period within a specific region and was based on a single field-sampling campaign. Therefore, the observed relationships primarily represent the stand conditions during the study period. Longer-term observations across additional stand ages and sites would help further evaluate the temporal stability and broader applicability of these density-associated patterns.

## 5 Conclusions

This study revealed contrasting density-associated patterns between aboveground tree growth and belowground carbon and nutrient storage in a *Picea crassifolia* plantation in the Qilian Mountains. The MPC and PPC treatments were associated with greater tree height, ground diameter, and crown development, whereas the AP and LPC treatments generally showed greater SOC, TN, and TP densities in several soil layers. These findings indicate that stand-density conditions favorable for individual-tree growth do not necessarily coincide with those associated with greater soil carbon and nutrient storage. Exploratory SEM further showed that soil TN emerged as the strongest statistical correlate of SOC among the measured soil variables, while soil water content, TP, and plant nutrient traits were also associated with SOC through direct or indirect model pathways. Overall, the results highlight the need to balance aboveground growth and belowground carbon and nutrient functions when considering density management. Under the conditions of this study, the relative advantages of AP and LPC for soil carbon storage provide a site-specific management hypothesis that should be further evaluated across different stand ages, sites, and longer temporal scales.

## Data Availability

The original contributions presented in the study are included in the article/supplementary material. Further inquiries can be directed to the corresponding author.
